# Molecular cloning and characterization of vanillin dehydrogenase from *Streptomyces* sp. NL15-2K

**DOI:** 10.1186/s12866-018-1309-2

**Published:** 2018-10-24

**Authors:** Motohiro Nishimura, Susumu Kawakami, Hideaki Otsuka

**Affiliations:** Department of Pharmaceutical Chemistry, Faculty of Pharmacy, Yasuda Women’s University, 6-13-1 Yasuhigashi, Asaminami-ku, Hiroshima, 731-0153 Japan

**Keywords:** Vanillin dehydrogenase, *Streptomyces*, Biotransformation, Salicylaldehyde dehydrogenase

## Abstract

**Background:**

*Streptomyces* sp. NL15-2K, previously isolated from the forest soil, features an extensive catabolic network for lignin-derived aromatic compounds, including pathways transforming ferulic acid to vanillin, vanillic acid, and protocatechuic acid. To successfully use *Streptomyces* sp. NL15-2K as a biocatalyst for vanillin production, it is necessary to characterize the vanillin dehydrogenase (VDH) that degrades the produced vanillin to vanillic acid, as well as the gene encoding this enzyme. Here, we cloned the VDH-encoding gene (*vdh*) from strain NL15-2K and comprehensively characterized its gene product.

**Results:**

The *vdh* open reading frame contains 1488 bp and encodes a 496-amino-acid protein with a calculated molecular mass of 51,705 Da. Whereas the apparent native molecular mass of recombinant VDH was estimated to be 214 kDa by gel filtration analysis, sodium dodecyl sulfate-polyacrylamide gel electrophoresis revealed a subunit molecular mass of ca. 56 kDa, indicating that VDH is a homotetramer. The recombinant enzyme showed optimal activity at 45 °C and pH 9.5. The VDH substrate specificity followed this order: vanillin (100%) > protocatechualdehyde (91%) > benzaldehyde (79%) > *p*-hydroxybenzaldehyde (56%) > isovanillin (49%) ≈ salicylaldehyde (48%) > anisaldehyde (15%) ≈ veratraldehyde (12%). Although peptide mass fingerprinting and BLAST searches indicated that this enzyme is a salicylaldehyde dehydrogenase (SALDH), the determined kinetic parameters clearly demonstrated that the enzyme is a vanillin dehydrogenase. Lastly, phylogenetic analysis revealed that VDH from *Streptomyces* sp. NL15-2K forms an independent branch in the phylogenetic tree and, hence, is evolutionarily distinct from other VDHs and SALDHs whose activities have been confirmed experimentally.

**Conclusions:**

Our findings not only enhance the understanding of the enzymatic properties of VDH and the characteristics of its amino acid sequence, but also contribute to the development of *Streptomyces* sp. NL15-2K into a biocatalyst for the biotransformation of ferulic acid to vanillin.

**Electronic supplementary material:**

The online version of this article (10.1186/s12866-018-1309-2) contains supplementary material, which is available to authorized users.

## Background

Lignin is a crosslinked phenylpropanoid polymer that is the most abundant component of plant biomass after cellulose. Partial decay of lignin yields numerous aromatic monomers that can be used in the cosmetics, food, pharmaceutical, and chemical industries. For example, vanillin is an essential aromatic compound extensively used as a flavoring agent in foods and beverages, and as an aromatic additive in perfumes, air fresheners, medicines, etc. The global vanillin demand exceeds 16,000 tons a year [1], and is expected to grow further in the future. Natural vanillin is extracted from the cured seedpods of *Vanilla planifolia*. However, the amount that can be supplied to the global market from this source is negligible because of the extremely high production cost. Therefore, most of the vanillin supplied to the market is chemically synthesized. However, together with the current increasing awareness of consumer health and safety, the demand for natural vanillin is growing.

**Fig. 1:**
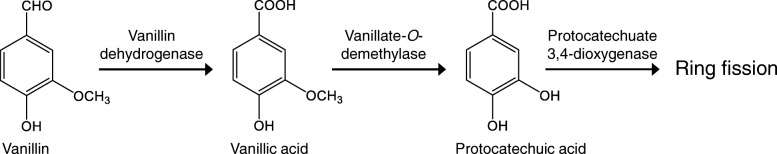
Degradation pathway of vanillin. The major pathway common to bacteria that degrade vanillin is shown

As an alternative to chemical synthesis of vanillin, biotransformation of plant constituents such as ferulic acid and eugenol to vanillin by various microbial species was proposed. Such microbial species include fungi, e.g., *Pycnoporus cinnabarinus* [2, 3], and bacteria, e.g., *Bacillus* [4, 5], *Pseudomonas* [6, 7], *Rhodococcus* [8], and *Streptomyces* [9], or recombinant *Escherichia coli* strains carrying genes encoding enzymes that mediate the biotransformation [10–14]. However, biotransformation to vanillin by using a whole-cell biocatalyst frequently entails a reduction in the vanillin yield either because of the toxicity of the highly reactive aromatic aldehyde group of vanillin or because of the degradation of vanillin to vanillic acid (Fig. 1). Methods employing resins to absorb the produced vanillin have been devised to increase the yield [2, 9, 14]. Further, the inactivation of *vdh*, which encodes vanillin dehydrogenase (VDH), has been demonstrated in *Amycolatopsis* sp. ATCC 39116 [15] and *Pseudomonas fluorescens* BF13 [6]. Although vanillin accumulation was markedly enhanced in both cases, a detectable amount of vanillic acid was generated and coupled to a slow degradation of vanillin; in these studies, it was proposed that vanillic acid was formed by a nonspecific aldehyde dehydrogenase (ALDH) or oxidase. Besides the biotransformation to vanillin, other intriguing approaches reported for vanillin production include a method involving the use of immobilized enzymes [16] and de novo biosynthesis from glucose [17].

Streptomycetes are gram-positive bacteria with a high G + C content of DNA. These bacteria are responsible for the production of the majority of antibiotics used worldwide. Most *Streptomyces* species are found in the soil, where they play an environmentally critical role in the recycling of organic materials because they have evolved complex and efficient enzyme systems that break down and catabolize diverse organic substances, including lignin and lignin-derived aromatic compounds. Therefore, *Streptomyces* species can potentially serve as biocatalysts for the production of valuable compounds, such as vanillin, from inexpensive plant constituents. Previously, we isolated *Streptomyces* sp. NL15-2K from a forest soil by screening for bacteria capable of catabolizing lignin-derived aromatic compounds [18]. Based on 16S rRNA gene phylogeny, this strain is most closely related to *Streptomyces capoamus* NBRC 13411. However, spore chain morphology (*Spira*-type) and spore surface ornamentation (spiny) of strain NL15-2K are clearly different from those (*Retinaculiaperti*-type; smooth) of the *S. capoamus* type strain. Analysis of the degradation products of lignin-derived aromatic compounds added to the medium as the sole carbon source revealed that this strain possesses an extensive catabolic network for diverse compounds, including the pathway transforming ferulic acid to vanillin [18]. Thus, we have been characterizing the enzymes, and their encoding genes, involved in the catabolism of lignin-derived aromatic compounds in *Streptomyces* sp. NL15-2K.

VDH, one of the key enzymes involved in the catabolism of lignin-derived aromatic compounds, degrades vanillin to vanillic acid. Although extensive studies of *vdh* genes have been performed [6, 15, 19–26], only a few studies reported both, the gene analysis and characterization of VDH enzyme [15, 25, 26]. No information for VDH from *Streptomyces* species is available. As noted above, *Streptomyces* sp. NL15-2K could potentially be used as a biocatalyst for vanillin production. However, to achieve this goal, it is essential to first characterize the VDH that degrades the produced vanillin and the encoding gene. Here, we isolated the *vdh* gene from *Streptomyces* sp. NL15-2K and comprehensively characterized the gene product.

## Methods

### Materials and chemicals

Vanillin (4-​hydroxy-​3-​methoxybenzaldehyde), vanillic acid (4-hydroxy-3-methoxybenzoic acid), ferulic acid (4-hydroxy-3-methoxycinnamic acid), isovanillin (3-​hydroxy-​4-​methoxybenzaldehyde), protocatechualdehyde (3,​4-​dihydroxybenzaldehyde), benzaldehyde, *p*-hydroxybenzaldehyde, *m*-anisaldehyde (3-​methoxybenzaldehyde), veratraldehyde (3,​4-​dimethoxybenzaldehyde), syringaldehyde (4-​hydroxy-​3,​5-​dimethoxybenzaldehyde), and salicylaldehyde (2-​hydroxybenzaldehyde) were purchased from Wako Pure Chemicals (Osaka, Japan). NAD(P)^+^ and lactate dehydrogenase from the rabbit muscle were purchased from Oriental Yeast Co. Ltd. (Osaka, Japan). Oligonucleotides were purchased from Life Technologies Japan Ltd. (Tokyo, Japan).

### Bacterial strains, vectors, and cultivation media

*Streptomyces* sp. NL15-2K used in this study was isolated from a forest soil in Canada and reported as a bacterium capable of degrading lignin-related aromatic compounds [18]. *Streptomyces* sp. NL15-2K was used for the purification of VDH and isolation of chromosomal DNA. The spores, formed on yeast extract-malt extract (YEME) medium (1% glucose, 0.5% polypeptone, 0.3% yeast extract, 0.3% malt extract, and 0.04% MgCl_2_·6H_2_O, pH 7.0) [27] supplemented with 1.5% agar, were inoculated and cultured at 30 °C in 100 mL of YEME medium. After 48 h in liquid culture, 10% of the grown mycelia were transferred to 100 mL of mineral salts medium with yeast extract (MSMYE; 0.01% (NH_4_)_2_SO_4_, 0.01% NaCl, 0.02% MgSO_4_·7H_2_O, 0.001% CaCl_2_, 0.05% KH_2_PO_4_, 0.1% K_2_HPO_4_, and 0.05% yeast extract, pH 7.2) [28] supplemented with 3.6 mM ferulic acid as the sole carbon source, and incubated for an appropriate time at 30 °C. To isolate chromosomal DNA, bacteria were cultured in YEME medium supplemented with 17% sucrose and 0.5% glycine. Chromosomal DNA was extracted as described previously [27]. *E. coli* DH5α (Takara Bio, Kyoto, Japan) and pMD20-T (Takara Bio) were used for cloning of PCR products and sequencing, and *E. coli* BL21(DE3) (Novagen, Darmstadt, Germany) and pET-28a(+) (Novagen) were used for protein expression. *E. coli* strains were cultured in Luria-Bertani (LB) broth or on LB agar supplemented with ampicillin (50 μL/mL) or kanamycin (30 μL/mL) when necessary.

### Assay for VDH activity

VDH activity was assayed using two methods. For assay method I, the activity was determined by monitoring the decrease in absorbance at 340 nm caused by the transformation of vanillin to vanillic acid. A reaction mixture (0.2 mL) containing 0.1 M sodium phosphate buffer (pH 7.0), 125 μM vanillin, 0.5 mM NAD^+^, 1.2 mM pyruvate, 5.0 U/mL lactate dehydrogenase, and the enzyme was incubated at 30 °C for 10 min. The residual vanillin concentration was determined spectrophotometrically at 340 nm; the molar absorption coefficients at 340 nm used for vanillin determination were 11,872 M^− 1^·cm^− 1^ at pH 7.0 and 22,320 M^− 1^·cm^− 1^ at pH 9.5. Enzyme activity with salicylaldehyde was also determined using assay method I, but the change in substrate amount was monitored at 400 nm and the molar absorption coefficient used was 5750 M^− 1^·cm^− 1^ at pH 9.5. For assay method II, enzyme activity was determined by quantifying the residual vanillin after incubation with the enzyme by using high-performance liquid chromatography (HPLC). The enzyme was incubated with the reaction mixture as above but lacking pyruvate and lactate dehydrogenase. The reaction was terminated by heating at 80 °C for 5 min, and 10-μL aliquot of the mixture was analyzed by HPLC by using a Kinetex 5 u C18 100A column (4.6 × 250 mm; Phenomenex Inc., Torrance, CA). Elution was performed using 30% methanol containing 0.1% phosphoric acid, at a flow rate of 1.0 mL/min at 45 °C, and the absorption at 220 nm was monitored. In both methods, 1 U of enzyme was defined as the amount of enzyme that oxidized 1 μmol of vanillin per minute. The specific activity was expressed as U/mg protein, where the protein concentration was determined by using a Bio-Rad Protein Assay kit (Bio-Rad, Hercules, CA) with bovine serum albumin as the standard. The optimal temperature and pH for enzyme activity, the effects of metal ions on activity, and substrate specificity were determined using assay method II. To determine the effect of metal ions on enzyme activity, a positive control incubated without metal ions was included for comparison. When investigating substrate specificity, the following standards were used (retention times are given in parentheses): vanillin (6.3 min), isovanillin (5.9 min), veratraldehyde (10.0 min), syringaldehyde (6.7 min), *m*-anisaldehyde (14.2 min), protocatechualdehyde (4.1 min), *p*-hydroxybenzaldehyde (5.6 min), salicylaldehyde (10.9 min), and benzaldehyde (11.2 min). The kinetic parameters of the recombinant VDH were determined from Lineweaver-Burk plots. All measurements were normalized to negative controls without the enzyme, and repeated at least three times.

### Purification of VDH from *Streptomyces* sp. NL15-2K cells and recombinant *E. coli* cells

*Streptomyces* cells (22 g) were harvested from 3 L of culture and washed once with buffer A (25 mM sodium phosphate buffer, pH 7.0) containing 1 mM EDTA and 1 M KCl and then twice with buffer A containing 1 mM EDTA and 1 mM phenylmethylsulfonyl fluoride. The washed cells were suspended in 50 mL of the same buffer, and disrupted for 5 min at 20 kHz using an ultrasonic disruptor (UD-201; TOMY, Tokyo, Japan). After the debris and unbroken cells were removed by centrifugation (30,000×*g*, 30 min), the resulting cell-free extract was loaded onto an ion-exchange column (2.1 × 21 cm, DEAE-Sepharose FF; GE Healthcare Japan, Tokyo, Japan) equilibrated with buffer A. Elution of proteins was carried out using a linear gradient of NaCl (0 to 1 M) in buffer A. VDH-containing fractions were pooled, dialyzed against buffer A containing 1 M ammonium sulfate using Slide-A-Lyzer G2 dialysis cassettes (10 K MWCO; Thermo Fisher Scientific, Waltham, MA), and loaded onto a RESOURCE PHE column (6 mL; GE Healthcare) equilibrated with the same buffer. VDH was eluted using a linear gradient of ammonium sulfate (1 to 0.2 M) in buffer A. The enzyme fractions were pooled, concentrated using an Amicon Ultra-15 centrifugal filter unit (molecular mass cutoff of 50,000 Da; Millipore, Bedford, MA), and passed through a Superdex 200 10/300 GL column (24 mL; GE Healthcare) equilibrated with buffer A containing 0.2 M NaCl. The enzyme fractions were pooled, dialyzed against buffer A, and loaded onto a Mono Q 4.6/100 PE column (1.7 mL; GE Healthcare) equilibrated with buffer A. VDH was eluted using a linear gradient of NaCl (0 to 0.4 M) in the buffer. The active fraction was analyzed by sodium dodecyl sulfate-polyacrylamide gel electrophoresis (SDS-PAGE) and peptide mass fingerprinting (PMF).

Recombinant VDH from a cell-free extract of *E. coli* cells (3.3 g) was purified by following the procedure described above, except that in the first purification step, the ion-exchange chromatography was performed using a HiPrep DEAE FF 16/10 column (20 mL; GE Healthcare), and the gel filtration step was omitted. The active fractions were pooled and stored at − 20 °C until use.

### Molecular mass determination

The molecular mass of the native VDH was determined using a Superdex 200 10/300 GL gel filtration column. The column was calibrated with thyroglobulin (669 kDa), ferritin (440 kDa), aldolase (158 kDa), conalbumin (75 kDa), and ovalbumin (44 kDa), all obtained from GE Healthcare. The subunit molecular mass of VDH was estimated by SDS-PAGE, using molecular size markers (EzStandard; ATTO, Tokyo, Japan) as reference proteins. Electrophoresis was performed using pre-cast 12.5% polyacrylamide gels (e-PAGEL; ATTO) under reducing conditions. Proteins were visualized using a Bio-Safe Coomassie Stain (Bio-Rad).

### Protein identification by PMF analysis

The protein band corresponding to the VDH subunit was excised from a stained SDS-PAGE gel and subjected to in-gel tryptic digestion (In-Gel Tryptic Digestion Kit, Thermo Fisher Scientific), according to the manufacturer’s protocol. Samples were prepared by mixing 1 μL of the peptide mixture with 0.5 μL of matrix solution (5 mg/mL α-cyano-4-hydroxycinnamic acid in 60% acetonitrile containing 0.1% trifluoroacetic acid). Peptide masses were determined by matrix-assisted laser desorption/ionization time-of-flight mass spectrometry (MALDI-TOF MS) performed on an Axima Performance mass spectrometer (Shimadzu, Kyoto, Japan), and the protein was identified using Mascot software (Matrix Science, London, UK).

### Cloning and sequencing of the *vdh* gene

Genes of four salicylaldehyde dehydrogenases (SALDHs) that shared high similarity with NL15-2K VDH according to PMF analysis were aligned as described in the Results. Based on the alignment, two degenerate primers, vdh-F (5′-ATGTCAGCYACTGAGAYCRMGGC-3′) and vdh-R (5′-TCAGATGGGGTAGTGGCGKGAYC-3′), were designed and used to amplify the *Streptomyces vdh* gene by PCR. PCR was performed using PrimeSTAR GXL DNA Polymerase (Takara Bio) with NL15-2K chromosomal DNA as the template under the following conditions: denaturation for 3 min at 96 °C; followed by 30 cycles of 1 min at 95 °C, 1 min at 60 °C, and 1 min at 72 °C; and a final extension for 10 min at 72 °C. The approximately 1.5-kb amplified product was inserted into pMD20-T vector by using the Mighty TA-cloning Reagent Set for PrimeSTAR (Takara Bio), and the nucleotide sequence was determined using an ABI 3730*xl* DNA Analyzer (Applied Biosystems, Foster City, CA) and a BigDye Terminator v3.1 Cycle Sequencing Kit (Applied Biosystems). The nucleotide and amino acid sequences were analyzed using GENETYX software ver. 11 (Genetyx, Tokyo, Japan). Database searches were performed using BLAST program available at the DDBJ website (https://www.ddbj.nig.ac.jp/index.html). Multiple amino acid sequences were aligned using ClustalW2.1 program [29]. The phylogenetic tree was inferred from the alignments by using the neighbor-joining method [30] and drawn using TreeView program [31]. The nucleotide sequence of the VDH-coding region was submitted to DDBJ/EMBL/GenBank (accession number LC383356).

### Heterologous expression of the *vdh* gene

The *vdh* coding sequence was PCR-amplified using a sense primer, 5′-AACCC**ATG**GCAGCCACTGAGACCG-3′ (*Nco*I site underlined), and an antisense primer, 5′-AACCTCGAGCC**TCA**GATGGGGTAGTGGCGG-3′ (*Xho*I site underlined). The sense and antisense primers were designed to introduce the ATG initiation codon and TGA stop codon (boldfaced) for VDH production, respectively. The amplified gene was digested with *Nco*I and *Xho*I, purified using a MinElute PCR Purification Kit (Qiagen, Hilden, Germany), and inserted into the respective restriction sites in pET-28a(+) (Novagen) to generate pET28a/VDH. After verifying the nucleotide sequence of *vdh* by sequencing, pET28a/VDH was used to transformed *E. coli* BL21(DE3), and the resulting recombinant strain was used for VDH production. *E. coli* BL21(DE3) harboring pET28a/VDH was cultured in LB broth supplemented with 1% glucose and kanamycin (30 μL/mL) at 37 °C. The overnight culture was diluted 1:100 in fresh LB broth supplemented with kanamycin (30 μL/mL) and incubated at 30 °C until the optical density at 600 nm reached 0.8. VDH production was induced by adding 1 mM isopropyl-β-D-thiogalactopyranoside (IPTG), and the cultures were incubated overnight at 30 °C. *E. coli* BL21(DE3) harboring the empty vector pET-28a(+) was used as a negative control strain.

## Results

### Purification of VDH from *Streptomyces* cells and PMF analysis

**Fig. 2:**
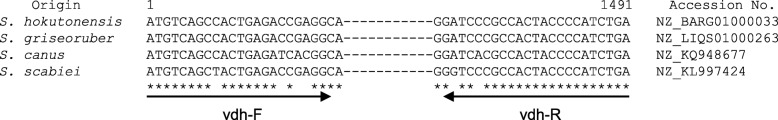
Nucleotide sequence alignment of salicylaldehyde dehydrogenases (SALDHs). Nucleotide sequences corresponding to N-terminal and C-terminal regions of SALDHs are shown. Asterisks: identical nucleotides; arrows: locations of degenerate primers used for *vdh* gene cloning

Endogenous VDH purified in four chromatography steps from cell-free extract of *Streptomyces* sp. NL15-2K migrated as a single protein band after SDS-PAGE (Additional file 1: Figure S1). However, purification of this enzyme was highly challenging, because the protein tended to become unstable with increasing purity, and because the growth rate of the bacteria was low. Therefore, we sought to use recombinant VDH for enzyme characterization. To start identifying the enzyme, the VDH protein band was analyzed by PMF. The analysis revealed a high similarity between the VDH purified from NL15-2K cells and SALDHs from other *Streptomyces* species. Mascot protein scores (scores > 83 were significant at *P* < 0.05) were 104, for SALDH from *S. hokutonensis*; 102, for SALDH from *S. canus*; and 92, for SALDHs from *S. scabiei* and *S. griseoruber*. The four SALDHs contained 496 amino acids and their amino acid sequences were almost identical (94–97% identity) (Additional file 2: Figure S2). Similarly, alignment of the nucleotide sequences of the genes encoding these SALDHs revealed a high level of nucleotide identity (92–96%). Based on the nucleotide sequences of the N-terminal and C-terminal regions of the proteins, two degenerate primers, vdh-F and vdh-R, were designed for PCR amplification of *vdh* from *Streptomyces* sp. NL15-2K (Fig. 2).

### Cloning and sequence analysis of the *vdh* gene

**Fig. 3:**
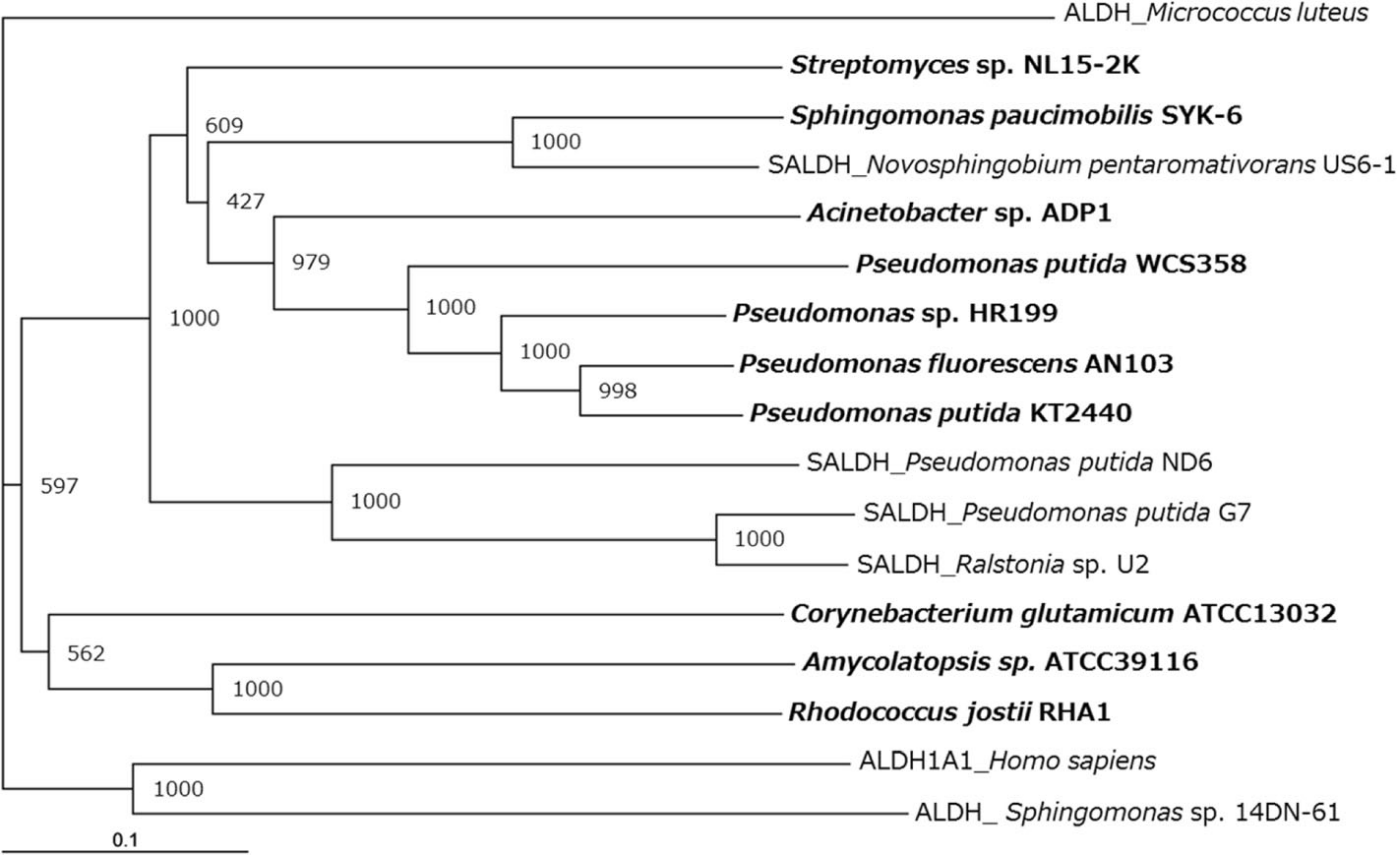
Phylogenetic tree based on amino acid sequences of VDHs, SALDHs, and aldehyde dehydrogenases (ALDHs). Bootstrap values are presented at the nodes [39]. Scale bar: evolutionary distance of 0.1 amino acid residue per position in the sequence. Boldface type: functionally verified VDHs. The SALDHs and ALDHs included here were also experimentally characterized. Accession numbers for the sequences are as follows: VDHs: *S. paucimobilis* SYK-6, BAF45815; *Acinetobacter* sp. ADP1, AAP78946; *P. putida* WCS358, CAA75076; *Pseudomonas* sp. HR199, O05619; *P. fluorescens* AN103, CAA73503; *P. putida* KT2440, NP_745497; *C*. *glutamicum* ATCC 13032, NP_601867; *Amycolatopsis* sp. ATCC 39116, JX292129; and *R. jostii* RHA1, WP_011595659; SALDHs: *Novosphingobium pentaromativorans* US6–1, AIT79348; *P. putida* ND6, AAP44246; *P. putida* G7, BAE92159; and *Ralstonia* sp. U2, AAD12613; and ALDHs: *Micrococcus luteus* NDB3Y10, KWW42988; *Homo sapiens*, NP_000680; and *Sphingomonas* sp. 14DN-61, BAE19973

The *vdh* gene was successfully PCR-amplified using the primers vdh-F and vdh-R. DNA sequencing revealed that the *vdh* open reading frame was 1488-bp long and encoded a 496-amino-acid protein with a calculated molecular mass of 51,705 Da, and an isoelectric point of 5.00. A BLAST search revealed that the deduced amino acid sequence was highly identical with those of proteins annotated as VDH from *S. scabiei* S58 (accession no. WP_059082879; 95% identity), ALDH from *S. bottropensis* ATCC 25435 (WP_005473480; 94% identity), and SALDHs from *Streptomyces* sp. Root 369 (WP_057613570; 94% identity) and *S. ciscaucasicus* DSM 40275 (WP_062046703; 94% identity), as well as the four SALDHs described above (93–95% identity). VDH from strain NL15-2K also shared high amino acid sequence identity with the functionally characterized VDHs from *Pseudomonas* sp. HR199 (O05619; 58% identity), *P. fluorescens* AN103 (CAA73503; 54% identity), and *P. putida* KT2440 (NP_745497; 53% identity). The *Pseudomonas* enzymes and NL15-2K VDH shared the ALDH glutamic acid active site, LELGGKAP (amino acids 263–270 in NL15-2K VDH) [32]. Two conserved ALDH amino acid residues, Cys-298 and K-186, were also conserved in NL15-2K VDH. Cys-298 is the catalytic cysteine, and is proposed to be responsible for mediating the dehydrogenase activity in cooperation with E-264 and K-186 [33]. A consensus motif, Gly-X-Lys-X-Ser-Gly-X-Gly (X = any amino acid), which is present near the carboxy terminus of several ALDHs and might represent a candidate coenzyme-binding site, was also identified in the C-terminal region (amino acids 461–468) of NL15-2K VDH, although the third residue in the motif, Lys, was replaced by Gly. To assess the phylogenetic relationship between NL15-2K VDH, other VDHs, SALDHs, and aromatic ALDHs whose enzyme activity has been verified, we aligned their amino acid sequences and constructed a phylogenetic tree (Fig. 3). The analysis revealed that VDH from *Streptomyces* NL15-2K clustered with all VDHs from gram-negative bacteria, including the three *Pseudomonas* VDH enzymes described above, but was located in a branch distinct from these VDHs. Intriguingly, VDH from *Streptomyces* sp. NL15-2K was located in a cluster distinct from that containing VDHs from the three gram-positive bacteria, *Corynebacterium glutamicum* ATCC 13032, *Amycolatopsis* sp. ATCC 39116, and *Rhodococcus jostii* RHA1, which indicated that *Streptomyces* VDH is evolutionarily different from these VDHs.

### Gene expression and purification of recombinant VDH

**Fig. 4:**
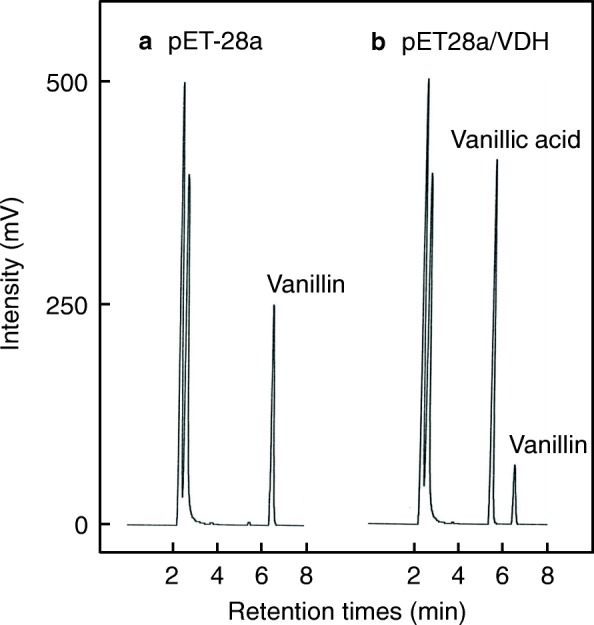
Conversion of vanillin to vanillic acid by VDH. HPLC analysis was used to examine vanillin conversion to vanillic acid by VDH activity in cell-free extracts. A cell-free extract of *E. coli* harboring pET-28a (**a**) or pET28a/VDH (**b**) was incubated with 1.25 mM vanillin in the presence of 2 mM NAD^+^ for 10 min. HPLC analysis was conducted according to the conditions described in Methods. *E. coli* strains were cultured for 15 h after IPTG induction

**Fig. 5:**
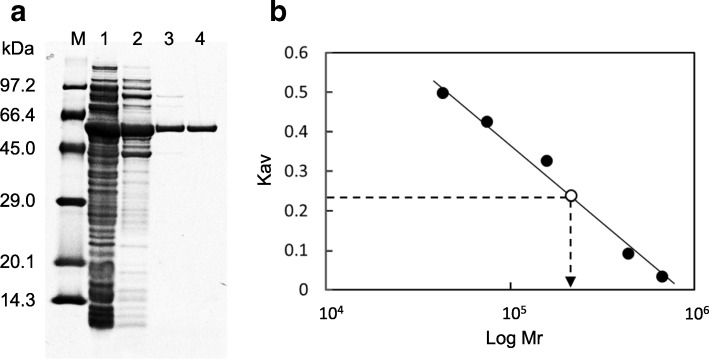
Molecular mass determination of VDH from *Streptomyces* sp. NL15-2K. **a** SDS-PAGE analysis of proteins from each purification step. Lane M: molecular mass markers (sizes indicated); lane 1: cell-free extract from *E. coli* harboring pET28a/VDH; lane 2: active fraction from HiPrep DEAE FF; lane 3: active fraction from RESOURCE PHE; lane 4: active fraction from Mono Q. **b** Native molecular mass determination of VDH through gel-filtration analysis. Open circle: VDH; closed circles: molecular mass standards

To express *vdh* in *E. coli*, the DNA sequence encoding *Streptomyces* VDH was amplified by PCR, cloned into the pET-28a(+) expression vector, and transformed into *E. coli*. VDH activity in cell-free extracts of recombinant *E. coli* was assayed by examining vanillin conversion to vanillic acid by HPLC (Fig. 4). As shown in Fig. 4b, the extract of the strain harboring pET28a/VDH indeed oxidized vanillin to vanillic acid. In contrast, the extract of the negative-control strain did not oxidize the substrate (Fig. 4a). These data demonstrate that the *vdh* gene was functionally expressed in *E. coli*. Next, we purified the recombinant VDH from a cell-free extract of *E. coli* harboring pET28a/VDH. Enhanced enzyme production in the heterologous host improved the yield of purified VDH; the results are summarized in Table 1. Recombinant VDH was purified 5.3-fold with 5% recovery, and 1.2 mg of purified enzyme was obtained from 0.5 L of *E. coli* culture. Figure 5a shows the SDS-PAGE profile of VDH preparations obtained at each purification step. Most protein contaminants were removed after two chromatography steps with HiPrep DEAE FF and RESOURCE PHE columns (Fig. 5a, lanes 2 and 3). The purified VDH migrated as a single band on a SDS-PAGE gel (Fig. 5a, lane 4).

**Table 1:** Purification of *Streptomyces* sp. NL15-2K VDH from the recombinant *E. coli*

Step	Total activity (U)	Total protein (mg)	Specific activity (U/mg)	Recovery (%)	Purification (fold)
Cell-free extract	42.1	133	0.32	100	1
Hiprep DEAE FF	19.6	32.8	0.60	47	1.9
RESOURCE PHE	4.7	3.5	1.34	11	4.3
Mono Q	2.0	1.2	1.66	5	5.3

### Molecular mass determination of VDH

The relative molecular mass of purified recombinant VDH was estimated by SDS-PAGE to be 56 kDa (Fig. 5a), i.e., roughly equivalent to the molecular mass calculated from the amino acid sequence. By contrast, the molecular mass of recombinant VDH determined by gel filtration on a Superdex 200 10/300 GL column was 214 kDa (Fig. 5b), which indicated that VDH from NL15-2K is a tetramer. This was in agreement with previous reports that VDHs from *P. fluorescens* BTP9 [34] and *Micrococcus* sp. TA1 [35] are tetrameric enzymes.

### The effect of pH and temperature on VDH activity

**Fig. 6:**
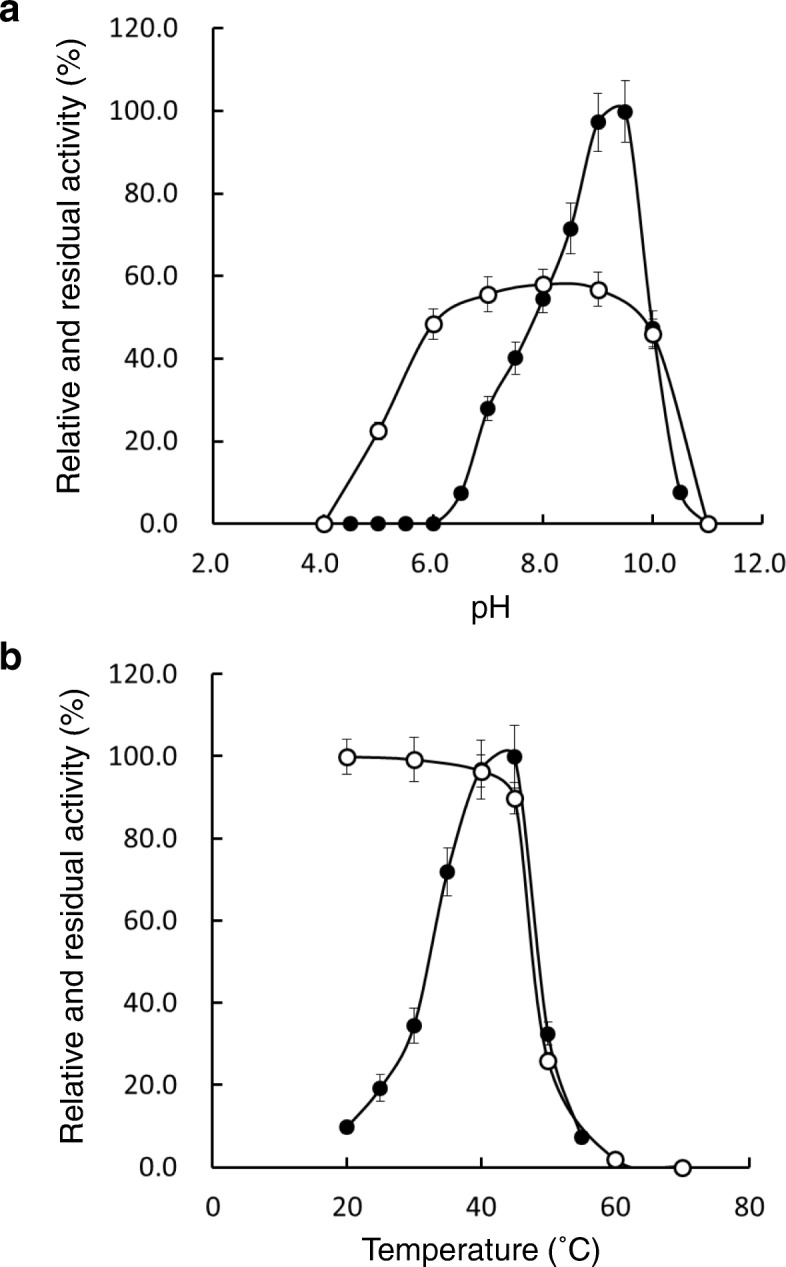
Effects of pH (**a**) and temperature (**b**) on VDH activity (●) and stability (○). The optimal pH was measured at 30 °C in buffer solutions of distinct pH under otherwise standard assay conditions. The buffer systems used were sodium acetate buffer (pH 4.0–5.5), sodium phosphate buffer (pH 6.0–7.5), Tris-HCl buffer (pH 8.0–9.0), and glycine-NaOH buffer (pH 9.5–11.0). The maximal activity, obtained at pH 9.5, was defined as 100%. The pH stability of VDH was determined by preincubating the enzyme in each buffer at 4 °C for 15 h and measuring the residual activity by using the standard assay, with the initial activity regarded as 100%. The optimal temperature was determined by measuring enzyme activity at various temperatures under otherwise standard assay conditions; the maximal activity, obtained at 45 °C, was defined as 100%. Lastly, thermal stability was determined by preincubating the enzyme at various temperatures for 10 min in sodium phosphate buffer (pH 7.0) and measuring the residual activity by using the standard assay method; the activity measured without preincubation was regarded as 100%. Data are shown as means ± standard deviation (error bars; n = 3)

We next examined how pH and temperature affect VDH activity and stability (Fig. 6). VDH from *Streptomyces* sp. NL15-2K exhibited an optimal pH and temperature of 9.5 and 45 °C, respectively, with high activity observed in narrow ranges of pH and temperature. The optimal pH of 9.5 was almost the same as that of VDHs from *P. fluorescens* BTP9 [34] and *Micrococcus* sp. TA1 [35]. On the other hand, the optimal pH reported for VDHs from *C. glutamicum* ATCC 13032 [25] and *Bacillus subtilis* 3NA [26] is 7.0, and that for VDHs from *Amycolatopsis* sp. ATCC 39116 [15] and *Burkholderia cepacia* TM1 [35] is 8.0. At all pH ranges tested, VDH lost > 40% of its initial activity after storage at 4 °C for 15 h, and the enzyme solution became clouded after storage at pH ≤ 5.0. To examine the VDH thermal stability, the enzyme was incubated at a temperature range of 20–70 °C for 10 min, and the residual activity was determined. VDH activity was retained up to 40 °C but declined sharply above 45 °C, and the enzyme solution incubated at 50 °C became slightly clouded. The observation of cloudiness of the enzyme solution indicated that the enzyme was readily denatured, which lowered its activity.

### The effect of metal ions on VDH activity

To assess the effect of metal ions on VDH activity, we prepared reaction mixtures containing specific ions, each at a final concentration of 1 mM. We tested eight metal ions (Ag^+^, Co^2+^, Cu^2+^, Fe^2+^, Mg^2+^, Mn^2+^, Zn^2+^, and Fe^3+^). The activity of VDH from *Streptomyces* sp. NL15-2K was partially inhibited by Zn^2+^ (85% of the initial activity was retained), markedly inhibited by Cu^2+^ (21% of the initial activity was retained), and completely inhibited by Ag^2+^. The other tested metal ions did not affect VDH activity.

### VDH substrate specificity

**Fig. 7:**
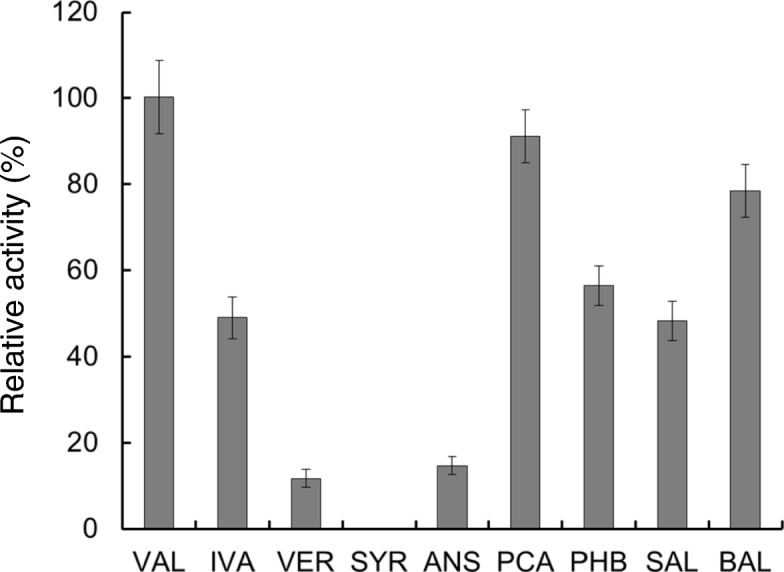
Substrate specificity. The substrate specificity of VDH was determined by measuring the enzyme activity at the optimal pH, 9.5, under otherwise standard assay conditions. The measured activity in each case is expressed here as the percentage of activity toward vanillin, and the data are shown as means ± standard deviation (error bars; n = 3). Abbreviations: VAL, vanillin; IVA, isovanillin; VER, veratraldehyde; SYR, syringaldehyde; ANS, anisaldehyde; PCA, protocatechualdehyde; PHB, *p*-hydroxybenzaldehyde; SAL, salicylaldehyde; BAL, benzaldehyde

The substrate specificity of VDH from *Streptomyces* sp. NL15-2K was evaluated by determining the enzyme’s catalytic activity with various aromatic aldehydes as substrates. This was done using assay method II, at the enzyme’s optimal pH (9.5), and the results are summarized in Fig. 7. The most specific substrate was vanillin (100% activity), followed by protocatechualdehyde (91%), benzaldehyde (79%), *p*-hydroxybenzaldehyde (56%), isovanillin (49%), salicylaldehyde (48%), anisaldehyde (15%), and veratraldehyde (12%). No activity with syringaldehyde was observed. We also investigated cofactor specificity of VDH toward NAD^+^ and NADP^+^. To do this, enzyme activity at the optimal pH (9.5) was determined in a reaction mixture containing NAD^+^ (0.25–2.5 mM) or NADP^+^ (0.25–10 mM). The kinetic parameters determined for NAD^+^ were as follows: *K*_m_*,* 647 ± 40.0 μM; *k*_cat_, 23.45 ± 0.59 s^− 1^; and *k*_cat_/*K*_m_, 0.036 ± 0.002 s^− 1^·μM^− 1^. By contrast, the kinetic parameters for NADP^+^ could not be determined because only a trace amount of enzyme activity was detected in the presence of NADP^+^. The observed cofactor specificity agrees with reports indicating that most VDH enzymes identified thus far preferentially use NAD^+^ [15, 23, 35]. However, the *K*_m_ of *Streptomyces* VDH was considerably higher than that of VDH from *C. glutamicum* ATCC 13032 (26.25 ± 3.22 μM) [25]. This might be because of a reduction of the enzyme’s affinity for the cofactor caused by the Lys-to-Gly replacement in the putative coenzyme-binding site described above. By contrast, the consensus motif in VDH from *C. glutamicum* ATCC 13032 is conserved, without any amino acid residue replacements.

## Discussion

VDH converts vanillin to vanillic acid and is one of the key enzymes required for the catabolism of lignin-derived aromatic compounds in certain soil bacteria. In the current study, we cloned and expressed the *vdh* gene from *Streptomyces* sp. NL15-2K, and functionally characterized the gene product. Prior to gene cloning, VDH was purified using *Streptomyces* cells grown in a medium containing ferulic acid as the sole carbon source, because VDH activity was not detected in cell-free extracts of cultures in YEME medium containing 1% glucose. Therefore, *Streptomyces* VDH is thought to be induced by ferulic acid. Similarly, as noted below, preliminary experiments indicated that the strain NL15-2K can use vanillin as the sole carbon source, which potentially suggests that vanillin also functions as an inducer of VDH expression. This is in agreement with a previous report that *vdh* expression in *Amycolatopsis* sp. ATCC 39116 appears to be induced by the enzyme’s substrate and structurally related compounds, such as ferulic acid [15]. Moreover, VDHs from *P. fluorescens* AN103 [36], *Micrococcus* sp. TA1 [35], and *B. cepacia* TM1 [35] are also reported to be substrate-inducible. In preliminary experiments, we found that when *Streptomyces* sp. NL15-2K was cultured in MSMYE containing vanillin as the sole carbon source, vanillin was first converted to vanillyl alcohol (4-hydroxy-3-methoxybenzyl alcohol) and then to vanillic acid, with higher amounts of the latter than of vanillyl alcohol (Additional file 2: Figure S3). This showed that NL15-2K harbors another pathway to avoid vanillin toxicity, where vanillin is reduced to vanillyl alcohol, in addition to the main pathway involving VDH. Similar observations were reported for *Amycolatopsis* sp. ATCC 39116 [37] and *B. subtilis* 3NA [26]. However, unlike these bacteria, which accumulate vanillyl alcohol, in *Streptomyces* sp. NL15-2K, vanillyl alcohol disappeared from the culture medium as cultivation progressed, which suggested that degradation via vanillyl alcohol might be an additional pathway for the detoxification and usage of vanillin. Three pathways of vanillyl alcohol degradation have been identified in the white-rot fungus *Lentinus edodes* [38]. Notably, the pathway that produces muconolactone via aromatic-ring oxidation and cleavage between positions C-3 and C-4 is the main route of vanillyl alcohol degradation in *L. edodes*, which may possibly also be involved in the degradation of vanillyl alcohol in *Streptomyces* sp. NL15-2K. In future studies, we will examine whether this strain harbors a pathway involving aromatic-ring cleavage of vanillyl alcohol.

*Streptomyces* VDH showed high substrate preference for vanillin, protocatechualdehyde, and benzaldehyde. According to reports published to date, VDHs can be classified into two groups based on substrate specificity. One group comprises enzymes showing the highest specificity for vanillin. The other group comprises enzymes that show higher specificity for aromatic aldehydes, such as *p*-hydroxybenzaldehyde, benzaldehyde, and isovanillin, than for vanillin. VDHs from *C. glutamicum* ATCC 13032 [25], *Amycolatopsis* sp. ATCC 39116 [15], *B. cepacia* TM1 [35], and *Sphingomonas paucimobilis* SYK-6 [23] belong to the first group, whereas VDHs from *Micrococcus* sp. TA1 and *B. subtilis* 3NA belong to the second group [26, 35]. VDH from *Micrococcus* sp. TA1 exhibits approximately 2.9- and 1.5-fold higher specificity for isovanillin and protocatechualdehyde, respectively, than for vanillin [35], and the specificity of VDH from *B. subtilis* 3NA for benzaldehyde is approximately 1.3-fold higher than that for vanillin [26]. Based on these observations, *Streptomyces* VDH is classified as a member of the first group of VDH enzymes. In terms of the relative cofactor specificity, VDHs from NL15-2K, *Amycolatopsis* sp. ATCC 39116, *B. cepacia* TM1, and *S. paucimobilis* SYK-6 showed markedly higher preference for NAD^+^ than NADP^+^. Thus, these enzymes clearly differ from *Corynebacterium* VDH, which uses both NAD^+^ and NADP^+^ with similar efficiency [25]. The optimal pH for *Streptomyces* VDH activity was determined to be 9.5, whereas pH 8.0 was found to be optimal for VDHs from *Amycolatopsis* sp. ATCC 39116 and *B. cepacia* TM1. Taken together, *Streptomyces* VDH is slightly different from the other VDHs. The specificities for vanillin and NAD^+^ of *Streptomyces* VDH are similar to those of *Sphingomonas* VDH, but these enzymes are somewhat different with respect to substrate specificity: when the activity toward vanillin was defined as 100%, the activities of *Streptomyces* VDH toward anisaldehyde and *p*-hydroxybenzaldehyde were 15% and 56%, respectively, whereas the corresponding activities of *Sphingomonas* VDH were 54% and 34% [23], respectively. Since no other features of *Sphingomonas* VDH have been reported, further comparison is difficult. Similarly, among VDHs closely related to NL15-2K VDH, based on the constructed phylogenetic tree (Fig. 3), VDHs from *Acinetobacter* and *Pseudomonas* strains have not been characterized, although their genes have been sequenced and the enzymes verified as VDHs. Therefore, their enzymatic properties cannot be compared with those for *Streptomyces* VDH.

BLAST search indicated that the amino acid sequence of VDH from *Streptomyces* sp. NL15-2K was highly identical to the sequences of certain proteins annotated as SALDHs from *Streptomyces* species. However, the VDH showed higher substrate specificity toward vanillin than salicylaldehyde (Fig. 7). Furthermore, the catalytic efficiency (*k*_cat_/*K*_m_) of *Streptomyces* VDH toward vanillin (1.11 ± 0.10 s^− 1^·μM^− 1^) was 43-fold higher than that toward salicylaldehyde (0.026 ± 0.001 s^− 1^·μM^− 1^), which indicated that vanillin was the preferred substrate (Table 2). This difference in catalytic efficiency is associated with the markedly higher affinity of VDH for vanillin (*K*_m_ = 8.47 ± 0.91 μM) than salicylaldehyde (*K*_m_ = 566 ± 81.5 μM). The turnover number for salicylaldehyde (*k*_cat_ = 14.91 ± 1.58 s^− 1^) was slightly higher than that for vanillin (*k*_cat_ = 9.35 ± 0.77 s^− 1^), but this appeared to be caused by a lower affinity of the enzyme for salicylaldehyde. These observations indicated that *Streptomyces* VDH should function as a VDH rather than as a SALDH. Among the VDHs whose functions have been clarified to date, kinetic parameters for vanillin have been reported only for VDH from *C. glutamicum* ATCC 13032, with the *K*_m_ of 26.31 ± 3.09 μM; *k*_cat_ of 21.03 ± 1.22 s^− 1^; and *k*_cat_/*K*_m_ of 0.80 ± 0.04 s^− 1^·μM^− 1^ [25]. These values were determined under the optimal assay condition for that enzyme. A comparison of the kinetic parameters of VDHs from *Streptomyces* sp. NL15-2K and *C. glutamicum* ATCC 13032 revealed that the catalytic efficiency of *Streptomyces* VDH was slightly higher than that of *Corynebacterium* VDH. Although the turnover number of *Corynebacterium* VDH is approximately two times higher than that of *Streptomyces* VDH, the *K*_m_ of *Streptomyces* VDH for vanillin was three times lower than that of *Corynebacterium* VDH. Consequently, the lower turnover number of *Streptomyces* VDH might be associated with a more robust affinity of the enzyme for vanillin.

**Table 2:** Kinetic parameters of VDH from *Streptomyces* sp. NL15-2K

Substrate	*K*_m_ (μM)	*k*_cat_ (s^−1^)	*k*_cat_/*K*_m_ (s^−1^·μM^−1^)	Catalytic efficiency ratio of vanillin to salicylaldehyde
Vanillin	8.47 ± 0.91	9.35 ± 0.77	1.11 ± 0.10	43
Salicylaldehyde	566 ± 81.5	14.91 ± 1.58	0.026 ± 0.001	1

## Conclusions

As shown in the current study, VDH from *Streptomyces* sp. NL15-2K shows high substrate specificity for vanillin, and then should play a major role in vanillin catabolism. We previously found that when the strain NL15-2K was grown in MSMYE supplemented with ferulic acid as the sole carbon source, ferulic acid was degraded to vanillic acid, without any vanillin detected [18]. This indicated that the vanillin formed was rapidly degraded during ferulic acid catabolism. Therefore, interruption of the two degradation pathways, proceeding via vanillic acid and via vanillyl alcohol, is indispensable for the use of *Streptomyces* sp. NL15-2K as a biocatalyst for vanillin production. To the best of our knowledge, this is the first report describing the enzymatic function and gene sequence of a *Streptomyces* VDH. This study not only enhances the understanding of the enzymatic properties of VDH and the characteristics of its amino acid sequence, but also contributes to the development of this *Streptomyces* strain into a biocatalyst for the biotransformation of ferulic acid to vanillin.

## Additional files

Additional file 1: **Figure S1.** SDS-PAGE of vanillin dehydrogenase (VDH) from *Streptomyces* sp. NL15-2K. Lane 1: molecular mass markers (sizes indicated); lane 2: active fraction from the final purification step (on a Mono Q column). (PDF 393 kb)

Additional file 2: **Figure S2.** Amino acid sequence alignment of SALDHs. Amino acid sequences of *Streptomyces* SALDHs, retrieved from Mascot database, were aligned using ClustalW program. The accession numbers for SALDH sequences from the four species are the following: *S. hokutonensis*, WP_019069277; *S. canus*, WP_059211011; *S. griseoruber*, WP_055635242; and *S. scabiei*, WP_037697438. Asterisks: identical amino acids. **Fig. S3.** Degradation of vanillin by *Streptomyces* sp. NL15-2K. Cells were cultured at 30 °C in MSMYE containing 3.6 mM vanillin as the sole carbon source. Closed circles: vanillin; triangles: vanillyl alcohol; squares: vanillic acid. Vanillin degradation was monitored by performing HPLC under the conditions described in Methods; the retention times of vanillin and its products were as follows: vanillin, 6.2 min; vanillic acid, 5.3 min; vanillyl alcohol, 3.8 min. (PDF 429 kb)

## Abbreviations

ALDH: Aldehyde dehydrogenase
HPLC: High-performance liquid chromatography
IPTG: Isopropyl-β-D-thiogalactopyranoside
MALDI-TOF MS: Matrix-assisted laser desorption/ionization time-of-flight mass spectrometry
MSMYE: Mineral salts medium with yeast extract
PMF: Peptide mass fingerprinting
SALDH: Salicylaldehyde dehydrogenase
SDS-PAGE: Sodium dodecyl sulfate-polyacrylamide gel electrophoresis
VDH: Vanillin dehydrogenase
YEME: Yeast extract-malt extract

## References

[1] Brochado AR, Matos C, Møller BL, Hansen J, Mortensen UH, Patil KR (2010). Improved vanillin production in baker's yeast through in silico design. Microb Cell Factories.

[2] Lesage-Meessen L, Lomascolo A, Bonnin E, Thibault JF, Buleon A, Roller M (2002). A biotechnological process involving filamentous fungi to produce natural crystalline vanillin from maize bran. Appl Biochem Biotechnol.

[3] Tilay A, Bule M, Annapure U (2010). Production of biovanillin by one-step biotransformation using fungus *Pycnoporus cinnabarinus*. J Agric Food Chem.

[4] Yan L, Chen P, Zhang S, Li S, Yan X, Wang N (2016). Biotransformation of ferulic acid to vanillin in the packed bed-stirred fermentors. Sci Rep.

[5] Paz A, Outeiriño D, Pinheiro de Souza Oliveira R, Domínguez JM (2018). Fed-batch production of vanillin by *Bacillus aryabhattai* BA03. N Biotechnol.

[6] Di Gioia D, Luziatelli F, Negroni A, Ficca AG, Fava F, Ruzzi M (2011). Metabolic engineering of *Pseudomonas fluorescens* for the production of vanillin from ferulic acid. J Biotechnol.

[7] Graf N, Altenbuchner J (2014). Genetic engineering of *Pseudomonas putida* KT2440 for rapid and high-yield production of vanillin from ferulic acid. Appl Microbiol Biotechnol.

[8] Plaggenborg R, Overhage J, Loos A, Archer JA, Lessard P, Sinskey AJ (2006). Potential of *Rhodococcus* strains for biotechnological vanillin production from ferulic acid and eugenol. Appl Microbiol Biotechnol.

[9] Hua D, Ma C, Song L, Lin S, Zhang Z, Deng Z (2007). Enhanced vanillin production from ferulic acid using adsorbent resin. Appl Microbiol Biotechnol.

[10] Achterholt S, Priefert H, Steinbüchel A (2000). Identification of *Amycolatopsis* sp. strain HR167 genes, involved in the bioconversion of ferulic acid to vanillin. Appl Microbiol Biotechnol.

[11] Overhage J, Steinbüchel A, Priefert H (2003). Highly efficient biotransformation of eugenol to ferulic acid and further conversion to vanillin in recombinant strains of *Escherichia coli*. Appl Environ Microbiol.

[12] Barghini P, Di Gioia D, Fava F, Ruzzi M (2007). Vanillin production using metabolically engineered *Escherichia coli* under non-growing conditions. Microb Cell Factories.

[13] Yamada M, Okada Y, Yoshida T, Nagasawa T (2008). Vanillin production using *Escherichia* coli cells over-expressing isoeugenol monooxygenase of *Pseudomonas putida*. Biotechnol Lett.

[14] Yoon SH, Lee EG, Das A, Lee SH, Li C, Ryu HK (2007). Enhanced vanillin production from recombinant *E. coli* using NTG mutagenesis and adsorbent resin. Biotechnol Prog.

[15] Fleige C, Hansen G, Kroll J, Steinbüchel A (2013). Investigation of the *Amycolatopsis* sp. strain ATCC 39116 vanillin dehydrogenase and its impact on the biotechnical production of vanillin. Appl Environ Microbiol.

[16] Furuya T, Kuroiwa M, Kino K (2017). Biotechnological production of vanillin using immobilized enzymes. J Biotechnol.

[17] Hansen EH, Møller BL, Kock GR, Bünner CM, Kristensen C, Jensen OR (2009). De novo biosynthesis of vanillin in fission yeast (*Schizosaccharomyces pombe*) and baker’s yeast (*Saccharomyces cerevisiae*). Appl Environ Microbiol.

[18] Nishimura M, Ooi O, Davies J (2006). Isolation and characterization of *Streptomyces* sp. NL15-2K capable of degrading lignin-related aromatic compounds. J Biosci Bioeng.

[19] Venturi V., Zennaro F., Degrassi G., Okeke B. C., Bruschi C. V. (1998). Genetics of ferulic acid bioconversion to protocatechuic acid in plant-growth-promoting Pseudomonas putida WCS358. Microbiology.

[20] Priefert H, Rabenhorst J, Steinbüchel A (1997). Molecular characterization of genes of *Pseudomonas* sp. strain HR199 involved in bioconversion of vanillin to protocatechuate. J Bacteriol.

[21] Overhage J, Priefert H, Rabenhorst J, Steinbüchel A (1999). Biotransformation of eugenol to vanillin by a mutant of *Pseudomonas* sp. strain HR199 constructed by disruption of the vanillin dehydrogenase (*vdh*) gene. Appl Microbiol Biotechnol.

[22] Plaggenborg R, Overhage J, Steinbüchel A, Priefert H (2003). Functional analyses of genes involved in the metabolism of ferulic acid in *Pseudomonas putida* KT2440. Appl Microbiol Biotechnol.

[23] Masai E, Yamamoto Y, Inoue T, Takamura K, Hara H, Kasai D (2007). Characterization of *ligV* essential for catabolism of vanillin by *Sphingomonas paucimobilis* SYK-6. Biosci Biotechnol Biochem.

[24] Chen HP, Chow M, Liu CC, Lau A, Liu J, Eltis LD (2012). Vanillin catabolism in *Rhodococcus jostii* RHA1. Appl Environ Microbiol.

[25] Ding W, Si M, Zhang W, Zhang Y, Chen C, Zhang L (2015). Functional characterization of a vanillin dehydrogenase in *Corynebacterium glutamicum*. Sci Rep.

[26] Graf N, Wenzel M, Altenbuchner J (2016). Identification and characterization of the vanillin dehydrogenase YfmT in *Bacillus subtilis* 3NA. Appl Microbiol Biotechnol.

[27] Hopwood DA, Bibb MJ, Chater KF, Kieser T, Bruton CJ, Kieser HM (1985). Genetic manipulation of *Streptomyces*: a laboratory manual.

[28] Chow KT, Pope MK, Davies J (1999). Characterization of a vanillic acid non-oxidative decarboxylation gene cluster from *Streptomyces* sp. D7. Microbiology.

[29] Larkin MA, Blackshields G, Brown NP, Chenna R, McGettigan PA, McWilliam H (2007). Clustal W and Clustal X version 2.0. Bioinformatics.

[30] Saitou N, Nei M (1987). The neighbor-joining method: a new method for reconstructing phylogenetic trees. Mol Biol Evol.

[31] Page RD (1996). TreeView: an application to display phylogenetic trees on personal computers. Comput Appl Biosci.

[32] PROSITE database. PS00687. https://prosite.expasy.org/.

[33] González-Segura L, Rudiño-Piñera E, Muñoz-Clares RA, Horjales E (2009). The crystal structure of a ternary complex of betaine aldehyde dehydrogenase from *Pseudomonas aeruginosa* provides new insight into the reaction mechanism and shows a novel binding mode of the 2′-phosphate of NADP^+^ and a novel cation binding site. J Mol Biol.

[34] Baré G, Swiatkowski T, Moukil A, Gerday C, Thonart P (2002). Purification and characterization of a microbial dehydrogenase: a vanillin:NAD(P)^+^ oxidoreductase. Appl Biochem Biotechnol.

[35] Mitsui R, Hirota M, Tsuno T, Tanaka M (2010). Purification and characterization of vanillin dehydrogenases from alkaliphile *Micrococcus* sp. TA1 and neutrophile *Burkholderia cepacia* TM1. FEMS Microbiol Lett.

[36] Narbad A, Gasson MJ (1998). Metabolism of ferulic acid via vanillin using a novel CoA-dependent pathway in a newly-isolated strain of *Pseudomonas fluorescens*. Microbiology.

[37] Fleige C, Meyer F, Steinbüchel A (2016). Metabolic engineering of the actinomycete *Amycolatopsis* sp. strain ATCC 39116 towards enhanced production of natural vanillin. Appl Environ Microbiol.

[38] Crestini C, Sermanni GG (1995). Aromatic ring oxidation of vanillyl and veratryl alcohols by *Lentinus edodes*: possible artifacts in the lignin peroxidase and veratryl alcohol oxidase assays. J Biotechnol.

[39] Felsenstein J (1985). Confidence limits on phylogenies: an approach using the bootstrap. Evolution.

