# Supplementary material for: Molecular cloning and characterization of vanillin dehydrogenase from Streptomyces sp. NL15-2K
Source: BMC Microbiol. 2018 Oct 24;18:154. doi: 10.1186/s12866-018-1309-2 (PMC6201588; doi:10.1186/s12866-018-1309-2)
Supplement: Supplementary file 1 — Figure S1. SDS-PAGE of vanillin dehydrogenase (VDH) from Streptomyces sp. NL15-2K. Lane 1: molecular mass markers (sizes indicated); lane 2: active fraction from the final purification step (on a Mono Q column). (PDF 393 kb) [file 12866_2018_1309_MOESM1_ESM.pdf]

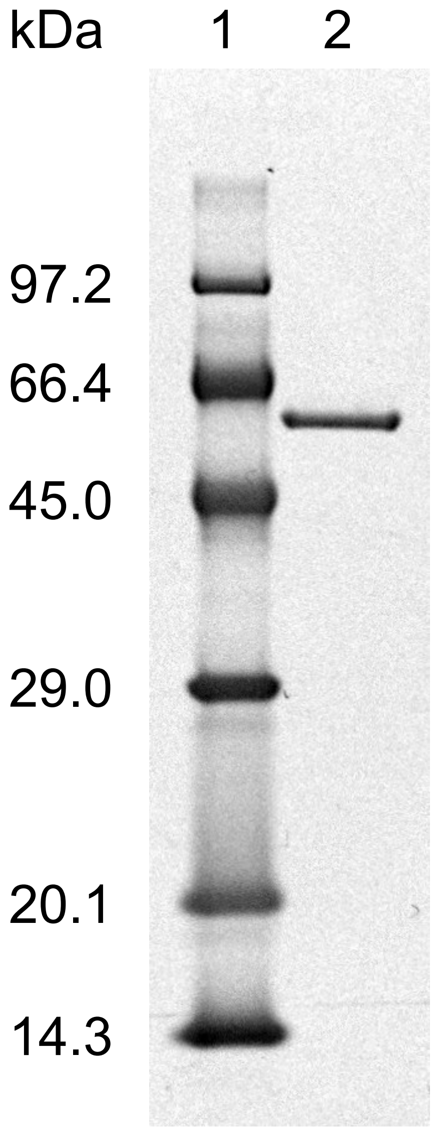

Additional file 1: Figure S1. SDS-PAGE of vanillin dehydrogenase (VDH) from *Streptomyces* sp. NL15-2K. Lane 1: molecular mass markers (sizes indicated); lane 2: active fraction from the final purification step (on a Mono Q column).
