# Supplementary material for: Molecular cloning and characterization of vanillin dehydrogenase from Streptomyces sp. NL15-2K
Source: BMC Microbiol. 2018 Oct 24;18:154. doi: 10.1186/s12866-018-1309-2 (PMC6201588; doi:10.1186/s12866-018-1309-2)
Supplement: Supplementary file 2 — Figure S2. Amino acid sequence alignment of SALDHs. Amino acid sequences of Streptomyces SALDHs, retrieved from Mascot database, were aligned using ClustalW program. The accession numbers for SALDH sequences from the four species are the following: S. hokutonensis, WP_019069277; S. canus, WP_059211011; S. griseoruber, WP_055635242; and S. scabiei, WP_037697438. Asterisks: identical amino acids. Fig. S3. Degradation of vanillin by Streptomyces sp. NL15-2K. Cells were cultured at 30 °C in MSMYE containing 3.6 mM vanillin as the sole carbon source. Closed circles: vanillin; triangles: vanillyl alcohol; squares: vanillic acid. Vanillin degradation was monitored by performing HPLC under the conditions described in Methods; the retention times of vanillin and its products were as follows: vanillin, 6.2 min; vanillic acid, 5.3 min; vanillyl alcohol, 3.8 min. (PDF 429 kb) [file 12866_2018_1309_MOESM2_ESM.pdf]

```
S. hokutonensis    1 MSATETEAPTSGAVRTSDLLIAGEYVAARDGRYFETTEALTGEPIARVAAASVEDVNLAVDAAAAALPEWSSLPPAARRSVLERAAVLLGERTNEIVATM 100
S. canus           1 MSATEITAPTSGAVRTSDLFIAGEHVAAKDGRYFETTEALTGEPIARVAAASVEDVNHAVDAAAAVLPEWSALPPAARRSVLERAAVLLGERTDEIVATM 100
S. griseoruber     1 MSATETEAPTSGAVRTSDLLIAGEHVAARDGRYFETTEALTGEPIARVAAASVEDVNLAVDAAAAALPEWSSLPPAARRSVLERAAVLLSERTDEIVATM 100
S. scabiei         1 MSATETEAPTSGTVRTSDLLIAGEHVAARDGRYFETTEALTGEPIARVAAASVEDVNLAVDAAAAALAEWSGLPPAARRSVLERAAVLLGERTDDIVATM 100
                     *****  ***** ****** **** *** **************************** ******* * *** ***************** ***  *****

S. hokutonensis  101 SREMGATLPWCGFNVHVAKGMFVEAAAQAYAAVGEVIPSDVPGLTALGVRQPVGVVVGIAPWNAPLILGVRAIVWPLVWGNTVVLKSSEQTPLTQAAIVR 200
S. canus         101 SREMGATLPWCGFNVHVAKGMFVEAAAQAYAAVGEVIPSDVPGLTALGVRQPVGVVVGIAPWNAPLILGVRAIVWPLVWGNTVVLKSSEQTPLTQAAIVQ 200
S. griseoruber   101 SREMGATLPWCGFNVHVAKGMFVEAAAQAYAAVGEVIPSDVPGLTALGVRQPVGVVVGIAPWNAPLILGVRAIVWPLVWGNTVVLKSSEQTPLTQAAIVQ 200
S. scabiei       101 SREMGATLPWCGFNVHVAKGMFVEAAAQAYSAVGEVIPSDVPGLTALGVRQPVGVVVGIAPWNAPLILGVRAIVWPLVWGNTVVLKSSEQTPLTQAAIVQ 200
                     ****************************** ********************************************************************

S. hokutonensis  201 VLHDAGVPAGAVNLISNAPQDGPSVVEALIAHPAVTRVNFTGSSKVGRIIGELGGRHLTRVVLELGGKAPFLVLPDADLEEAAAAASFGAFMNQGEICMS 300
S. canus         201 VLHDAGVPAGAVNLISNAPKDGPSVVEALIAHPAVTRVNFTGSSRVGRVIGELGGRHLTRVVLELGGKAPFLVLPDADLEEAAAAASFGAFMNQGEICMS 300
S. griseoruber   201 VLHDAGVPAGAVNLISNAPQDGPSVVEALIAHPAVTRVNFTGSSKVGRIIGELGGRHLTRVVLELGGKAPFLVLPDADLEEAAAAASFGAFMNQGEICMS 300
S. scabiei       201 VLHDAGVPAGAVNLISNAPEDGPGVVEALIAHPAVTRVNFTGSSRVGRIIGELGGRHLTRVVLELGGKAPFLVLPDADLEEAAAAASFGAFMNQGEICMS 300
                     ******************* *** ******************** *** ***************************************************

S. hokutonensis  301 TERVIVNRTVADELSSRLAERAAKLVVGPPSDPSSQIGPLVHAAAREHVVALIDDARAKGAQVLTGGTADGLFVQPTVLRGVTPDMRVYSEESFGPVVSI 400
S. canus         301 TERVIVDRTVADELSSRLAERASKLVVGPPSDPTSQIGPLVHGAARDHVVALIEDARAKGAQVLAGGTADGLFVQPTVLRGVTPDMRVYSEESFGPVVSI 400
S. griseoruber   301 TERVIVDGTVADELSSRLAERAAKLVVGPPSDPASQIGPLVHAAARDHVVALIEDARAKGAQVLTGGTADGLFVQPTVLRGVTPDMRIYSEESFGPVVSI 400
S. scabiei       301 TERVIVDRTVADELSSRLAERAAKLLVGPPSDPASQIGPLVHSAARDHVIELIEDARAKGAQVLTGGTADGLFVKPTVLRGVTPEMRVYGEESFGPLVSI 400
                     ******  ************** ** ******* ******** *** **  ** ********** ********* ********* ** * ****** ***

S. hokutonensis  401 IEVDSTDEAVAVANDTEYGLSAAVFGKDAAAALDVARRIRSGICHINGATVHDEPQMPFGGVGASGWGRFGSRAALEEFTELRWITIQSGSRHYPI     496
S. canus         401 IEVDSTDEAVAVANDTEYGLSAAVFGRDAAAALDVARRIRSGICHINGATVHDEPQMPFGGVGASGWGRFGSRAALEEFTELRWITIQSGSRHYPI     496
S. griseoruber   401 IEVDSTDEAVAVANDTEYGLSAAVFGKDAAAALDVARRIKSGICHINGATVHDEPQMPFGGVGASGWGRFGSRAALEEFTELRWITIQSGSRHYPI     496
S. scabiei       401 IEVDSTDEAVAVANDTEYGLSAAVFGRDAAAALDVARRIRSGICHINGATVHDEPQMPFGGVGASGWGRFGSRAALEEFTELRWITIQSGSRHYPI     496
                     ************************** ************ ********************************************************
```

Additional file 2: Figure S2. Amino acid sequence alignment of SALDHs. Amino acid sequences of *Streptomyces* SALDHs, retrieved from Mascot database, were aligned using ClustalW program. The accession numbers for SALDH sequences from the four species are the following: *S. hokutonensis*, WP_019069277; *S. canus*, WP_059211011; *S. griseoruber*, WP_055635242; and *S. scabiei*, WP_037697438. Asterisks: identical amino acids.

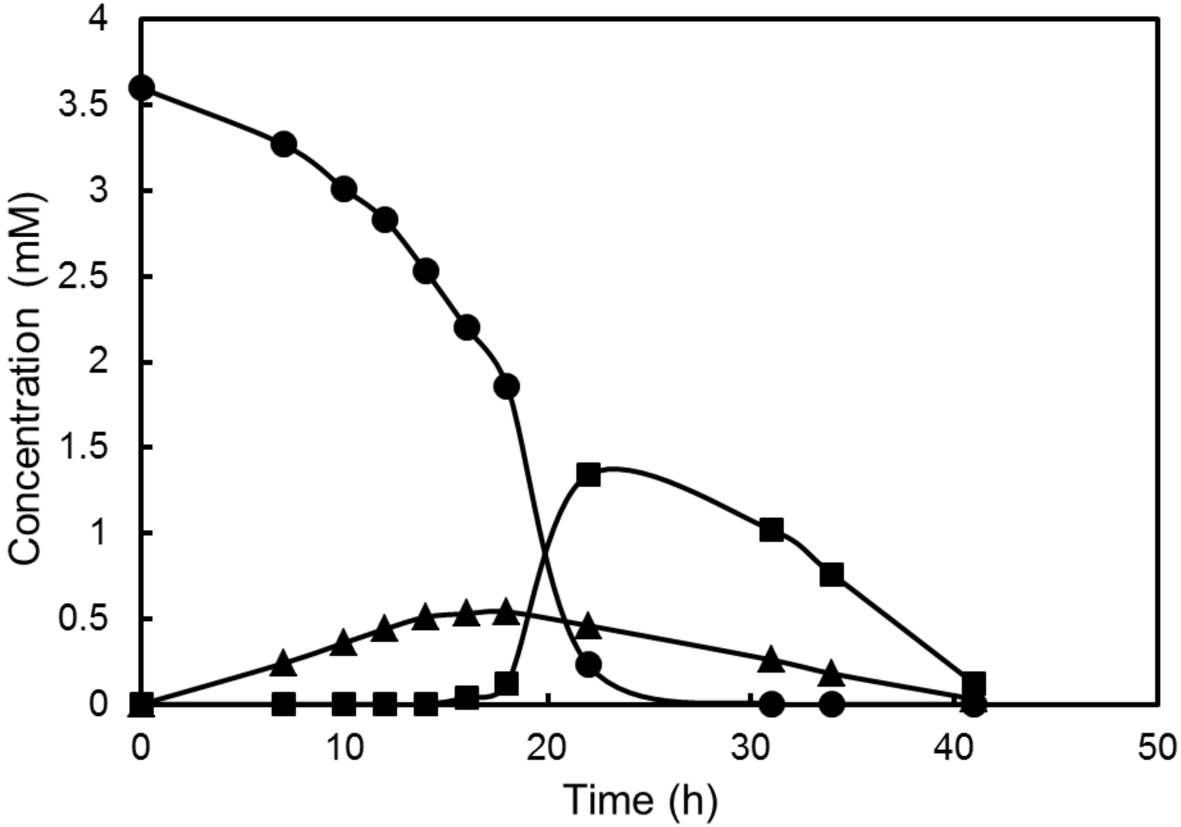

Additional file 2: Figure S3. Degradation of vanillin by *Streptomyces* sp. NL15-2K. Cells were cultured at 30°C in MSMYE medium containing 3.6 mM vanillin as the sole carbon source. Closed circles: vanillin; triangles: vanillyl alcohol; squares: vanillic acid. Vanillin degradation was monitored by performing HPLC under the conditions described in Methods; the retention times of vanillin and its products were as follows: vanillin, 6.2 min; vanillic acid, 5.3 min; vanillyl alcohol, 3.8 min.
